# Role of the Ubiquitin-Proteasome Systems in the Biology and Virulence of Protozoan Parasites

**DOI:** 10.1155/2015/141526

**Published:** 2015-05-19

**Authors:** Christian Muñoz, Juan San Francisco, Bessy Gutiérrez, Jorge González

**Affiliations:** Molecular Parasitology Unit, Medical Technology Department, Faculty of Health Sciences, University of Antofagasta, P.O. Box 170, Antofagasta, Chile

## Abstract

In eukaryotic cells, proteasomes perform crucial roles in many cellular pathways by degrading proteins to enforce quality control and regulate many cellular processes such as cell cycle progression, signal transduction, cell death, immune responses, metabolism, protein-quality control, and development. The catalytic heart of these complexes, the 20S proteasome, is highly conserved in bacteria, yeast, and humans. However, until a few years ago, the role of proteasomes in parasite biology was completely unknown. Here, we summarize findings about the role of proteasomes in protozoan parasites biology and virulence. Several reports have confirmed the role of proteasomes in parasite biological processes such as cell differentiation, cell cycle, proliferation, and encystation. Proliferation and cell differentiation are key steps in host colonization. Considering the importance of proteasomes in both processes in many different parasites such as *Trypanosoma, Leishmania, Toxoplasma,* and *Entamoeba*, parasite proteasomes might serve as virulence factors. Several pieces of evidence strongly suggest that the ubiquitin-proteasome pathway is also a viable parasitic therapeutic target. Research in recent years has shown that the proteasome is a valid drug target for sleeping sickness and malaria. Then, proteasomes are a key organelle in parasite biology and virulence and appear to be an attractive new chemotherapeutic target.

## 1. Introduction 

In a paper published in 1978, Ciehanover et al. [1] reported the presence of a heat-stable polypeptide component of an ATP-dependent proteolytic system isolated from reticulocytes. A second paper from the same researchers reported that the ATP-dependent conjugation of reticulocyte proteins to the polypeptide was required for protein degradation [2]. Based on these findings, Hershko et al. [3] proposed in 1980 that the ligation of ubiquitin to proteins targets them for degradation by a protease that specifically acts on proteins with several ubiquitin molecules attached [3, 4]. A “protease,” the 26S proteasome, was discovered by Hough and colleagues, who reported the identification and characterization of an ATP-dependent protease from rabbit reticulocyte lysates [5]. The real impact of this discovery would be dimensioned in the coming decades. In fact, Hershko's work on the ubiquitin enzymes was not only relevant but contributed to opening a new research field that was obscure and unexplored at that time. The 2004 Chemistry Nobel Prize award, conferred to Hershko, Ciechanover, and Rose “for the discovery of ubiquitin-mediated protein degradation,” was not only a recognition of these researchers but a recognition of the importance of the ubiquitin-proteasome pathway to the life of the cell and to health, disease, infection, and immunity [6]. Many researchers have contributed to our current knowledge of this biological pathway. Many relevant reviews have been already published. In this context, the main goal of this review is to attract attention to a new role of proteasomes: the biology and virulence of protozoan parasites. General aspects of the ubiquitin-proteasome pathways and inhibitors will be only summarized.

## 2. The Ubiquitin-Proteasome System

The bulk of the turnover of intracellular proteins in eukaryotic cells is carried out by two self-contained proteolytic systems, the lysosomes and proteasomes. Most proteins are degraded by the ubiquitin-proteasome system (UPS) [6].

Proteasomes are large complexes that perform crucial roles in many cellular pathways by degrading proteins in the cytosol and nucleus of eukaryotic cells to enforce quality control and regulate many basic cellular processes. Among these processes are progression through the cell cycle, signal transduction, cell death, immune responses, metabolism, protein-quality control, and development, in which proteasomes degrade short-lived regulatory or structurally aberrant proteins [6, 7]. The catalytic heart of these complexes, the 20S proteasome, is highly conserved in bacteria, yeast, and humans [8], with simpler versions also found in some* Archaea* and prokaryotes.

The 20S proteasome is a barrel-shaped assembly of 28 protein subunits. It forms a packed particle, a result of axial stacking of two outer *α* rings and two inner *β* rings made up of seven structurally related *α* and *β* subunits; the rings form an *α*_1–7_ *β*_1–7_ *β*_1–7_ *α*_1–7_ structure. The three subunits of each inner ring contain catalytically active threonine residues at their N termini and show N-terminal nucleophile hydrolase activity, indicating that the proteasome is a threonine protease [8]. The *β*1, *β*2, and *β*5 are associated with caspase-like, trypsin-like, and chymotrypsin-like activities, respectively, which confer the ability to cleave peptide bonds at the C-terminal side of acidic, basic, and hydrophobic amino acid residues, respectively. Those bonds that follow glycine and proline are less easily cleaved [9]. As revealed by structural studies performed by Huber and colleagues [10, 11], the potentially catastrophic elimination of inappropriate substrates is prevented by sequestration of active sites within the hollow structure of the 20S proteasome. Substrates access the central catalytic chamber through axial ports in the end rings of *α* subunits [12], although in the absence of activators, these channels are closed and proteasome activity is repressed. The 20S proteasome processively degrades client proteins, generating oligopeptides ranking in length from 3 to 15 amino acids. The resulting peptide products are subsequently hydrolyzed to amino acids by oligopeptidases and/or amino-carboxy peptidases [9].

Proteasomes are activated by protein complexes that bind to the end rings of *α* subunits. The best-known activator is PA700 [proteasome activator MW 700, also known as 19S or regulatory complex (RC)], which has been highly conserved from yeast to humans and binds to the 20S proteasome to form the 26S proteasome. PA700 is the only proteasome activator, that is, known to stimulate degradation of protein substrates. Thus, PA700 is thought to mediate most of the biological effects of the proteasome by facilitating substrate degradation [13, 14]. In contrast to PA700, two other evolutionarily conserved protein complexes that have been shown to bind specifically to and activate 20S proteasomes against model peptide substrates, PA28 (also known as 11S or REG) [7, 15] and PA200 [7, 16], do not recognize ubiquitinated proteins or use ATP. Proteasome activator PA200 enhances proteasome-mediated cleavage after acidic residues* in vitro*; however, in response to ionizing radiation, PA200 forms hybrid proteasomes with 19S caps and 20S core proteasomes that accumulate on chromatin, leading to an increase in proteolytic activity. A unique role for PA200 in genomic stability, that is, likely mediated through its ability to enhance post-glutamyl cleavage by proteasomes, has been reported [17]. Blm10/PA200 (*Saccharomyces cerevisiae*/human) does not utilize ATP and is generally believed to stimulate the hydrolysis of peptides but not proteins. Blm10/PA200 has been proposed to function in a surprisingly broad variety of processes [18], including 20S proteasome assembly [19], DNA repair [20], genomic stability [17], proteasome inhibition [21], spermatogenesis [22], and mitochondrial checkpoint regulation [23]. However, endogenous inhibitors like Hsp 90, P131, PR 39, and Tat have also been described. The biological role of 26S proteasomes and its activators and inhibitors have been reviewed extensively elsewhere [5, 7, 24, 25]. New regulatory mechanisms have emerged. The archaeal PAN ATPase complex is homologous to the eukaryotic 19S ATPases and contains a conserved C-terminal hydrophobic-tyrosine-X motif (HbYX), that is, essential for PAN to associate with the 20S proteasomes and open its gated channel for substrate entry [26]. Gate opening can be induced by C-terminal peptides from the 19S ATPase subunits, Rpt2, and Rpt5, but not by C-terminal peptides from PA28/26, which lack the HbYX motif and cause gate opening by distinct mechanisms. C-terminal residues in the 19S ATPases were also shown to be critical to the gating and stability of 26S proteasomes. Thus, the C termini of the proteasomal ATPases function like a “key in a lock” to induce gate opening and allow substrate entry [26]. Recently, it has been shown that binding of polyUb substrates to the 19S regulator stabilizes gate opening of the 20S proteasome and induces conformational changes in the 20S proteasome that facilitate channeling of substrates and their access to active sites. In consequence, polyUb substrates allosterically stimulate their own degradation, enhancing the peptidase activities of the 20S proteasome about two-fold in a process requiring ATP hydrolysis [27]. In addition, a recently published body of evidence suggests that many proteasome functions, such as substrate recognition, deubiquitylation, unfolding, and degradation, appear to be controlled allosterically [28, 29].

In this pathway, proteins are targeted for degradation by covalent ligation with ubiquitin. Ubiquitination tags the target protein with ubiquitin-like proteins (UBLs), such as ubiquitin, small ubiquitin-like modifier (SUMO), and NEDD8. Ubiquitination is a posttranslational modification of proteins in which the modifier is a polypeptide conjugated to the target proteins by an isopeptide bond between proteasome substrates: the C terminus of ubiquitin and one or more lysine side chains in the target proteins [30]. Protein modification by ubiquitin occurs in three successive steps that are mediated by three enzymes: the activating enzyme E1, the conjugating enzyme E2, and the ubiquitin ligase E3. This modification is reversible, and ubiquitinated proteins can be proteolytically deubiquitinated by specific deubiquitinating enzymes [30, 31]. Ubiquitin molecules can form polyubiquitin chains that are conjugated to target proteins, which are usually recognized and degraded by the proteasome [30, 32]; however, current knowledge of UPS strongly suggests that protein ubiquitination appears to be necessary but not essential. A recent paper reports that proteasomes can degrade a significant proportion of cellular proteins independent of ubiquitination. Then, 26S proteasomes specifically recognize and cleave similar sites, independent of ubiquitination, suggesting that disordered regions likely constitute the universal structural signal for proteasome-substrate proteolysis by proteasomes. In the same way, the inactivation of ubiquitin-activating enzyme E1 does not prevent intrinsic proteasome substrates degradation [33].

**Figure 1:**
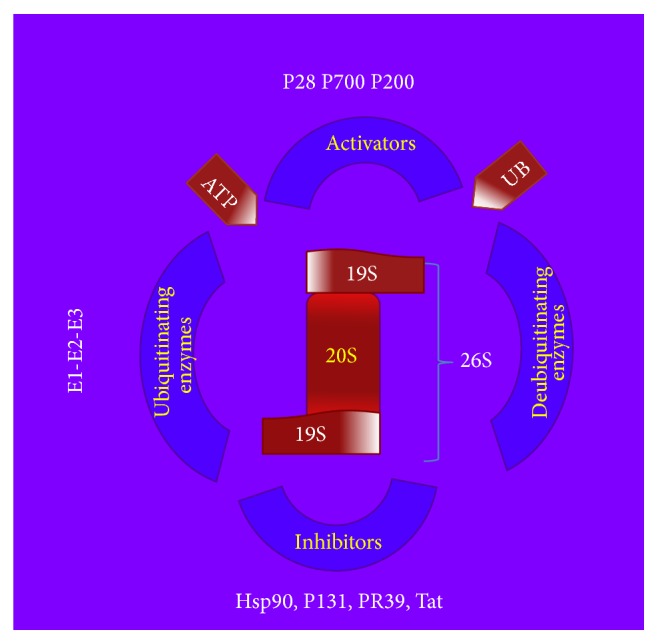
An overview of the main component of ubiquitin proteasome system.

The picture is completed by the deubiquitinating enzymes (DUBs) [30, 34]. They generate free Ub moieties from their initial translation products, recycle ubiquitin during breakdown of the poly-ubiquitin-protein conjugates, and/or reverse the effects of ubiquitination. All DUBs tested have remarkable specificity for ubiquitin. DUBs have been implicated in a variety of processes in animals and yeast, suggesting that individual DUBs are target-specific [34]. An intriguing possibility is that some DUBs can also regulate a protein's half-life by reversing ubiquitination. A large number of genes encode deubiquitinating enzymes, suggesting that many have highly specific and regulated functions. Interestingly, many of these enzymes are localized to subcellular structures or to molecular complexes. These localizations play important roles in determining functional specificity and can have major influences on their catalytic activities [34]. Indeed, recent findings strongly suggest that ubiquitination is regulated by both specific pathways of ubiquitination and deubiquitination. In summary, the protein substrates are first conjugated to multiple molecules of ubiquitin and then ubiquitin substrates are rapidly hydrolyzed by the 26S proteasome, an ATP-dependent complex comprising the core 20S proteasome enclosed by two proteasome activator (19S) regulatory complexes. Deubiquitination enzymes recycle the ubiquitin molecules and the pathway is modulated by protein activators and inhibitors. An overview of the ubiquitin-proteasome system is shown in Figure 1.

In summary, the ubiquitin-proteasome pathway is not only a degradation machine focused to destroy old or damaged proteins. This pathway is a major control point for regulating, among other things, short-lived proteins functioning as regulatory factors in a large array of cellular processes like cell-cycle progression [35], cell growth, stage-specific gene transcription [36], inflammatory response [37], and antigen processing [32]. Eukaryotic 20S proteasomes have several peptidase activities, as well as endoribonuclease, protein-chaperone, and DNA-helicase activities [38].

Until a few years ago, the role of proteasomes in parasite biology was completely unknown.

## 3. The Role of Proteasomes in Parasite Biology and Virulence

Parasitic protozoan are unicellular but complex cells that undergo multiple differentiation events to accommodate the various hosts and physical environments that they encounter in their life cycles.

Some proteases are involved in the differentiation of the infectious stages of a small number of protozoan parasites into their respective disease-causing stages [39–41]. The central role played by the proteolytic activities of the proteasome/ubiquitin system in regulating cell homeostasis has been demonstrated in a large number of fungi and higher eukaryotes and, more recently, in protozoan parasites [42].

A prominent feature of the life cycle of pathogenic parasites is the profound morphological changes they undergo during development in the vertebrate and invertebrate hosts. These developmental changes, during which shape, size, and cytoskeletal structures must adapt to the new stage, involve extensive and carefully controlled proteolysis. The intriguing question of what proteolytic system is involved in protein degradation in parasites led us to investigate the role of proteasomes in differentiation of protozoan parasites.

The protozoan parasites' 20S proteasomes are similar in morphology and size to the 20S proteasome isolated from archaebacteria, yeast, and mammals. Similarly, the composition of the protozoan proteasomes subunits is very similar to that of the eukaryotic proteasomes, with multiple *α* and *β* subunits instead of the single type of *α* and *β* subunit described in* Archaebacterias proteasomes*. Studies in different laboratories and with different models have found that inhibition of proteasomal function inhibits specific stages of morphological differentiation in* Trypanosoma*,* Plasmodium,* and* Entamoeba* and replication of* Plasmodium*,* Toxoplasma*,* Leishmania*, and* Trypanosoma*; however, invasion of the host cell is not inhibited in* Trypanosoma*,* Plasmodium*,* Leishmania,* or* Toxoplasma*. Differences between the proteasomes of mammals and parasites have been observed (*Trypanosoma*), as have differences in immunoreactive structures (*Trypanosoma* and* Toxoplasma*) and enzymatic activities (*Trypanosoma* and* Entamoeba*). These differences suggest that protozoan parasite proteasomes could be considered as a chemotherapeutic target, even though this organelle is also present in all host eukaryotic cells. In mammals cells, the ubiquitin proteasome system is essential for all eukaryotic cells; any alteration to its components thus has potential pathological consequences [43]. Chemotherapy targeting parasite proteasomes could result in successful therapies.

The following are the main findings reported in the literature with respect to the role of protozoan parasite proteasomes.

### 3.1. *Cryptosporidium*

A DNA sequence composed of 1281 nucleotides (nt) consisting of a single open reading frame (ORF) encoding a putative 20S proteasome *β*1-type subunit was isolated from* Cryptosporidium parvum*. Southern-blot analysis suggested that the sequenced DNA exists in the* C. parvum* genome as a single copy. The predicted protein consists of 210 amino acids (aa), including characteristic amino acids common to all proteasomal subunits, and is more similar to the beta1-type subunit of yeast than to other types of beta subunits [44]. No studies have examined its biological role.

### 3.2. *Giardia*

The parasite has a single gene that encodes monoubiquitin; however, two-dimensional electrophoresis assays have shown that the* Giardia* 20S proteasome seems to be as complex as that of other eukaryotes [45]. A study of the seven genes that encode the *α* subunits of the* G. duodenalis* proteasome indicated that the *α*-proteasome gene family evolved quickly from a single gene in the Archaea to seven or more genes in Eukarya [46]. The* G. duodenalis* 20S proteasome appears to be similar to that described in eukaryotic cells, containing a divergent set of *α* subunits [47]. Proteomics approaches performed to discover novel proteins associated with the stage-specific, Golgi-like encystation-specific vesicles (ESV) identified cytoplasmic and luminal factors of the endoplasmic reticulum quality-control system, that is, several structural (*α*) and catalytic (*β*) proteasome subunits. In contrast, cytoplasmic proteasome complexes undergo a developmentally regulated relocalization to ESVs during encystation. In mammalian cells and in yeast, proteasome complexes localize at ER membranes in addition to the cytoplasm and the nucleoplasm. Confocal microscopy analysis demonstrated that the giardial 20S core complex and 19S cap structure were associated with ESV membranes during early encystation until at least 7 h after induction. As noted previously, the expression of proteasome subunits is not upregulated in encysting cells [47]. The confocal microscopy data indicated a relocalization from more peripheral sites in the cytoplasm to the vicinity of ESVs, indicating a high rate of retrotranslocation of organelle proteins destined for degradation. In light of these results, the authors proposed that proteasome recruitment during encystation is a consequence of quality control and cargo maturation processes in the ER and early ESVs (i.e., protein folding, heterooligomerization, and trimming) producing large amounts of material destined for degradation [48].

### 3.3. *Entamoeba*

The 20S activity in proteasomes was described based on the SDS-electrophoretic pattern and immunoblot analysis of a soluble* Entamoeba histolytica* extract fractionated by density-gradient centrifugation [49]. A study of the* E. histolytica* proteome confirmed the presence of ubiquitin-proteasome components [50]. On the other hand, the genes encoding the *α* proteasome subunits show a higher identity with mammalian proteasomes (60.1% homology with rat proteasomes and 60.5% with human proteasomes) than with proteasomes from* Thermoplasma acidophilum* and* Saccharomyces cerevisiae* (39.5% and 53.8%, resp.). In* E. histolytica* trophozoites, nuclear localization of the 20S complex was not evident even by high-resolution confocal microscopy [51]. Instead, fluorescent reactivity against the proteasome subunits EhotS and EhS2 was observed exclusively in the cytosol, exhibiting a homogeneous distribution with no apparent exclusion of compartments that resemble the ER and Golgi apparatus, as observed in other cell types [30, 51].

Recently, multiple* E. histolytica* ubiquitination components, including ubiquitin and its activating (E1), conjugating (E2), and ligating (E3) enzymes, have been cloned and characterized. EhUbiquitin is activated by and forms a thioester bond with EhUba1 (E1)* in vitro* in an ATP- and magnesium-dependent fashion. According to the authors, EhUba1 exhibits a greater maximal initial velocity of pyrophosphate-ATP exchange than its human homolog, suggesting that different kinetics of ubiquitin activation might exist in* E. histolytica* [52].

In a reptilian amoeba,* Entamoeba invadens*, encystation is inhibited by lactacystin, a specific and irreversible inhibitor of proteasomes [53]; however, lactacystin seems to have no effect on* E. invadens* excystation [54].

### 3.4. *Leishmania*

These parasites are protozoan parasites with an intracellular stage called the amastigote that replicates in mammalian macrophages and an extracellular stage called the promastigote that replicates in the intestine of hematophagous insect belonging to genus* Lutzomyia*.

Purified proteasomes from* L. mexicana* were studied using polyacrylamide-gel electrophoresis (SDS-PAGE), revealing 10 different bands with masses ranging between 22 and 32 kDa, suggesting a complexity similar to that of eukaryotic proteasomes [55]. Lactacystin affected* L. mexicana* replication only when used at concentrations higher than 5 *μ*m, while MG132 blocked the same process at lower concentrations. These discrepancies might be due to the lower capacity of* L. mexicana* to incorporate these inhibitors. According to Christensen et al. [56], a new antigen that resembles an *α* subunit of the human 20S proteasome was identified in* Leishmania*. This antigen (LePa) is immunogenic in humans. Moreover, a DNA vaccine based on the LePa antigen induced an initial reduction in the size of lesions when mice were challenged with* Leishmania major*. The strong immunogenicity of the* Leishmania* proteasome was confirmed by Couvreur et al. [57], who reported that Antigen 24, an immunogenic complex isolated from* Leishmania infantum* used as reference antigen in the immunodiagnostic of human visceral leishmaniasis, was recognized by the serum of rabbits immunized with purified* L. mexicana* proteasomes. On the other hand, the* Leishmania chagasi* proteasome was partially purified and showed sensitivity to lactacystin and clasto-lactacystin beta-lactone, which blocked the* in vitro* growth of the promastigote stage. Although pretreatment of the promastigotes with lactacystin did not inhibit cell invasion, proteasomal function seems to be essential for replication and intracellular survival of amastigotes in the host cell [58].

In synchronized* Leishmania *cultures, Dubessay et al. [59] reported the cell-cycle-dependent regulation of protein levels. A kinesin called LmjKIN13-1 is highly abundant in the G2 + M phase and present at very low levels after mitosis. This protein is degraded through ubiquitin-proteasome pathways, demonstrating that it has C-terminal redundant degradation signals. This observation suggests that in* Leishmania*, in which postranduccional regulation is rare or absent, the proteasome appears to be involved in the regulation of protein levels [59]. On the other hand, in* Leishmania donovani, *degradation of pteridine reductase 1 (PTR1) has been reported. In* Leishmania*, PTR1 is an essential enzyme in pterin and folate metabolism. Western-blot studies using* L. donovani* promastigotes transfected with PTR1-GFP showed that PTR1 was degraded in the stationary phase of growth, when parasites start metacyclogenesis. Similarly, a probable destruction box composed of nine amino acids (Q63ADLSNVAK71) and a lysine K156 residue (as a site of ubiquitin conjugation) were identified in* L. donovani* PTR1. This finding suggests that degradation of PTR1 during the stationary phase of growth is mediated by proteasomes, resulting in low levels of H4-biopterin, which promotes metacyclogenesis and subsequently results in highly infective parasite stages [60]. Two HIV-protease inhibitors, indinavir and saquinavir, have been shown to block proteasome functions; effects were observed on the growth of* L. major* and* Leishmania infantum*. After 24 h of treatment, both drugs exhibited dose-dependent antileishmanial activity, with lethal-dose values of 50% (LD50), 8.3 *μ*M and 7 *μ*m on* L. major*, and minor activity on* L. infantum*. These results suggest the potential use of these protease inhibitors against opportunistic infections in treated seropositive patients [61].

It has also been reported in* L. donovani* that the proteasome is involved in downregulation of methionine adenosyltransferase (MAT), an enzyme important for metabolic processes; its product, S-adenosylmethionine (AdoMet), plays a key role in trans-methylation, trans-sulfuration, and polyamine synthesis. The presence of proteasome inhibitors such as MG-132, MG-115, epoxomicin, and lactacystin in the culture medium prevented MAT degradation in both MAT-overexpressing and “mock-transfected” leishmanial strains. The role of the ubiquitin (Ub) pathway in MAT downregulation was also supported by immunoprecipitation experiments. Immunoprecipitated MAT cross-reacted with anti-Ub antibodies, providing evidence of a proteasome-mediated downregulation of the leishmanial MAT abundance [62].

### 3.5. Trypanosomes

The* Trypanosoma* proteasome is the most intensely studied of the parasite proteasomes. The proteasome of* Trypanosoma brucei* was the first to be purified and characterized; however, its role in the biology of the parasite was not described [63]. Subsequent work showed that proteasome activity appears to be essential for cell-cycle progression, although participation seems to differ between the blood and procyclic forms. The amount of lactacystin needed to inhibit proliferation of procyclic forms concentrations was 5–10 *μ*m, five times higher than was needed to inhibit the same process in blood forms. According to the authors, this difference in sensitivity to inhibitors could be explained by differences in the cell permeability. DNA analysis by flow cytometry showed that in the procyclic forms, lactacystin inhibits the progression of the cell cycle in the G2 and M phases, while in blood forms, makes it in G1/S, G2, and M phases. According to the same authors, in* T. brucei,* lactacystin at 1 *μ*M was unable to block the differentiation of blood forms to the procyclic stage [64]. These results suggest that in trypanosomes, proteasomes participate in the regulation of cyclin levels [65]. The 20S proteasome purified from procyclic and bloodstream forms has increased trypsin-like activity, unlike the eukaryotic proteasomes in which the chymotrypsin-like activity is higher. In addition, other differences between* T. brucei* and mammalian proteasomes have been found. (1) The 20S proteasome of trypanosomes has a molecular weight of 630 kDa, while that of mammals is 700 kDa; (2) the 2D gels from the trypanosome 20S proteasome have only 26 protein spots, fewer than observed in the 20S proteasomes isolated from rat livers [66]; (3) although the morphology and size of the* T*.* brucei *proteasome are similar to those described in mammalian proteasomes, the pore diameter of the* T. brucei* 20S proteasome is greater than that observed in the rat 20S proteasome; (4) polyclonal antibodies raised against the human 20S proteasome cross-reacted with the procyclic and bloodstreams forms of* T. brucei* 20S proteasome; however, they strongly recognized rat 20S proteasome. On the other hand, polyclonal antibodies obtained against the purified 20S proteasome isolated from blood forms of* T. brucei* 20S also reacted with the purified 20S proteasome isolated from procyclic forms of the parasite but not with the 20S proteasome from rat erythrocytes. The *α*5 subunit of* T. brucei* proteasome has only 50% sequence identity with that of the rat proteasome [67].

A 20S proteasome activator was also identified in procyclic and blood forms of* T. brucei*.* In vitro,* the 26 kDa PA26 spontaneously polymerizes with proteasome 20S to generate the activated 20S proteasome [68]. Its human counterpart, PA28*α*, was as effective as PA26 in associating with and stimulating the enzymatic activity of the rat 20S proteasome but was unable to activate the proteasome 20S of* T. brucei*. Moreover, unlike mammalian and yeast proteasomes, the* T. brucei* proteasome is unable to degrade the mammalian ornithine decarboxylase-antizyme (ODC) complex, which catalyzes the first step in polyamine biosynthesis. This inability is a significant difference between trypanosomes and mammalian proteasomes [69]. Moreover, the functional characterization of 11 non-ATPase subunits (regulatory particles not-ATPase (Rpn)) in the 19S regulatory complex showed that when Rpn10 was deficient, a complex without Rpn was formed, but cell growth stopped. This structural dispensability but functional indispensability of Rpn10 constitutes another unique aspect of the* T. brucei* proteasome [70]. Similarly, proteomics and bioinformatic approaches have allowed the identification and mapping of* T. brucei *proteasomes [71].

Nine vinyl ester tripeptides selective for inhibition of mammalian proteasome trypsin-like activity have been tested for* in vitro* activity against* T. brucei*. Two showed trypanocidal activity in the low-micromolar range without displaying cytotoxicity against human cells; however, the compounds did not inhibit the trypsin-like activity of the trypanosome proteasome, although their effect correlates with inactivation of chymotrypsin-like activity. This finding suggests that the inhibitor sensitivities differ between mammalian and trypanosome proteasome. This difference may be exploited for rational antitrypanosomal drug development [72].

On the other hand, the role of the* T. cruzi* proteasomes in trypomastigote-to-amastigote differentiation has been clearly documented [73, 74]. Lactacystin significantly blocked the transformation of trypomastigotes to amastigotes in axenic medium at pH 5.0 [73]. The 20S proteasome was purified and characterized and shown to possess trypsin-like, chymotrypsin-like, and caspase-like activities. Treatment with lactacystin does not block cell invasion but strongly reduced discharge of the parasite. Similarly, leucine C^14^ metabolic labeling of trypomastigotes showed that proteolysis occurs during* T. cruzi* cell differentiation from trypomastigote to amastigote. This proteolytic pathway was blocked by proteasome inhibitors like lactacystin and vinyl sulphone, but not by serine or cysteinyl proteinase inhibitors, suggesting that the protein degradation that occurs during the parasite cell differentiation is proteasome-dependent [74]. Then, during parasite cell differentiation at acidic pH, an ATP-dependent proteolytic pathway was observed and 26S proteasomes were identified and characterized by first time in a protozoan parasite [74]. Similarly, these authors demonstrated that cytoskeleton proteins, especially the paraflagellar rod antigen, were degraded by a proteasome-dependent pathway. However, monoclonal antibodies raised against the* T. cruzi* 20S proteasome have been observed by electron microscopy and confocal studies, the presence of proteasomes in the nucleus, cytoplasm, and kinetoplasts. These findings were confirmed by detection of proteasome chymotrypsin-like activities in the kinetoplast, isolated by Percoll gradients [75]. In mammalian cells, the UPS has been found in the outer mitochondrial membrane associated degradation (OMMAD) quality controls proteins localized to the OMM [76]. Then, at the outer membrane, the UPS may play a role in recycling either membrane-embedded or imported proteins [77]. The role that proteasomes fulfill in* T. cruzi* kinetoplast is still unknown. However, we could speculate that proteasomes may be involved not only in quality control of proteins but also in kinetoplast morphology changes that occur when trypomastigotes differentiate to amastigotes or epimastigotes differentiate to metacyclic trypomastigotes.

According to Cardoso et al. [78], inhibition of the ubiquitin-proteasome pathway in* T. cruzi* epimastigotes does not block adhesion but does disrupt cell division. In the same way,* in vitro T. cruzi *metacyclogenesis was strongly inhibited (95%) by treatment with 5 *μ*M of lactacystin.

Proteasomal proteolysis during the* in vitro* metacyclogenesis of* T. cruzi* has also been studied. Cardoso et al. [79] demonstrated that proteasome-dependent proteolysis occurs during metacyclogenesis. No peaks of ubiquitin-mediated degradation were observed, and the profile of ubiquitinated conjugates was similar at all stages of differentiation; however, an analysis of carbonylated proteins showed significant variation in the levels of oxidized protein at the various stages of differentiation, and proteasome inhibition also increased oxidized-protein levels. These observations suggest that different proteasome complexes coexist during metacyclogenesis. The 20S proteasome may be free or linked to regulatory particles (PA700, PA26, and PA200), at specific cell sites, and the coordinated action of these complexes would make it possible for proteolysis of ubiquitin-tagged proteins and oxidized proteins to cooccur in the cell. In addition, these findings strongly suggest that the coordinated series of biochemical adaptations occurring during* T. cruzi* metacyclogenesis may also be regulated by the activity of different proteasome complexes. These data also highlight the importance of ubiquitin-independent proteasomal degradation during metacyclogenesis [79]. The role of proteasomes in cell differentiation led us to propose this organelle as a trypanosome virulence factor [80].

Two genes encoding the *α*1 and *α*6 subunits of the* T. cruzi *proteasome have been cloned and characterized [81]. Considering that the most part structural studies have been performed in trypanosomes [67, 69, 74, 75], a subunits composition of human, yeast, and trypanosomes is shown in Table 1.

### 3.6. *Plasmodium*

The presence of the* Plasmodium* proteasome was first shown using inhibitors. Lactacystin inhibits the* in vitro* development of exoerythrocytic forms of* Plasmodium berghei* but does not inhibit sporozoite invasion of the host cell. The inhibitory effect of lactacystin is stage-specific, and although no infected rat survived, lactacystin reduced the parasitemia of the infected animals. The authors thus suggested the proteasome as a promising chemotherapeutic target [82]. On the other hand, lactacystin inhibited the growth of three different lines of* Plasmodium falciparum* at similar molar concentrations and was more effective against chloroquine-resistant parasites [83]. The genes encoding the *β* subunits of* P. falciparum* 20S proteasome have been already cloned [84].

Phosphoethanolamine methyltransferase (fepm), an enzyme of central importance in the serine decarboxylase phosphoethanolamine methyltransferase (SDPM) pathway, is negatively transcriptionally regulated and degraded by the proteasome in the presence of choline. Immunoblotting, pulse-chase, and chromatin immunoprecipitation experiments have shown that Pfpmt degradation occurred not only in wild-type cells but also in transgenic parasites that express Pfpmt constitutively. The proteasome inhibitor bortezomib blocked choline-mediated Pfpmt degradation. These data were the first evidence that a metabolite can mediate transcriptional regulation and proteasome degradation in* Plasmodium* [85].

Gliotoxin (GTX), a metabolite of fungal origin, may have an* in vitro* antimalaria effect. GTX showed activity against chloroquine-sensitive and -resistant strains of* P. falciparum*. GTX cytotoxicity was significantly lower against normal liver cell lines [86]. According to the same researchers, GTX blocked chymotrypsin-like activity in the* P. falciparum* proteasome. In the same way, MLN-273, a proteasome inhibitor belonging to the peptidyl boronic acid family, has shown to inhibit the early intraerythrocytic stages of* P. falciparum,* as well as the exoerythrocytic stagesof* P. berghei*. The inhibitor did not affect the erythrocytes or the liver cells but caused a significant reduction in parasite protein degradation. According to these authors, the use of proteasome inhibitors as antineoplastic drugs suggests the possibility of malaria chemotherapy based on proteasome inhibitors [87]. From this perspective, proteasome inhibitors like bortezomib (Velcade: [(1R)-3-methyl-1-[[(2S)-1-oxo-3-phenyl-2-[(pyrazinylcarbonyl) amino] propyl] amino] butyl] boronic acid), which has been approved for the treatment of patients with myeloma, and a similar boronate called Z-Leu-Leu-Leu-B (OH) 2 (ZL3B), were assessed against four strains of* P. falciparum* (3D7, HB3, W2, and Dd2) which had different levels of sensitivity to antimalaria drugs like pyrimethamine and chloroquine. Both drugs were equally effective against susceptible and resistant parasites, blocking intraerythrocytic parasite development. These data strongly suggest that these drugs could be used alone or in association with malaria chemotherapy [88].

A comprehensive study of proteasome inhibitors active against* P. falciparum* laboratory strains and field isolates from Gabon showed that epoxomicin was highly active against* P. falciparum* and showed no signs of cross-resistance with similar drugs or any other proteasome inhibitor in an area with high-grade chloroquine resistance [89].

Although the* Plasmodium* proteasome has been suggested as potential antimalarial drug target, the toxicity of inhibitors has prevented validation of this enzyme* in vivo*. A screen of a library of 670 analogs of the recent US Food and Drug Administration-approved inhibitor, carfilzomib, was performed to identify compounds that selectively kill parasites. One of them, PR3, displayed significant parasite-killing activity* in vitro* but dramatically reduced toxicity in host cells. According to the authors, this parasite-specific toxicity was not due to selective targeting of the* Plasmodium* proteasome over the host proteasome but due to a lack of activity against one of the human-proteasome subunits. Subsequently, they used PR3 to significantly reduce parasite load in* P. berghei* infected mice without host toxicity, thus validating the proteasome as a viable antimalarial-drug target [90].

### 3.7. *Toxoplasma*

**Figure 2:**
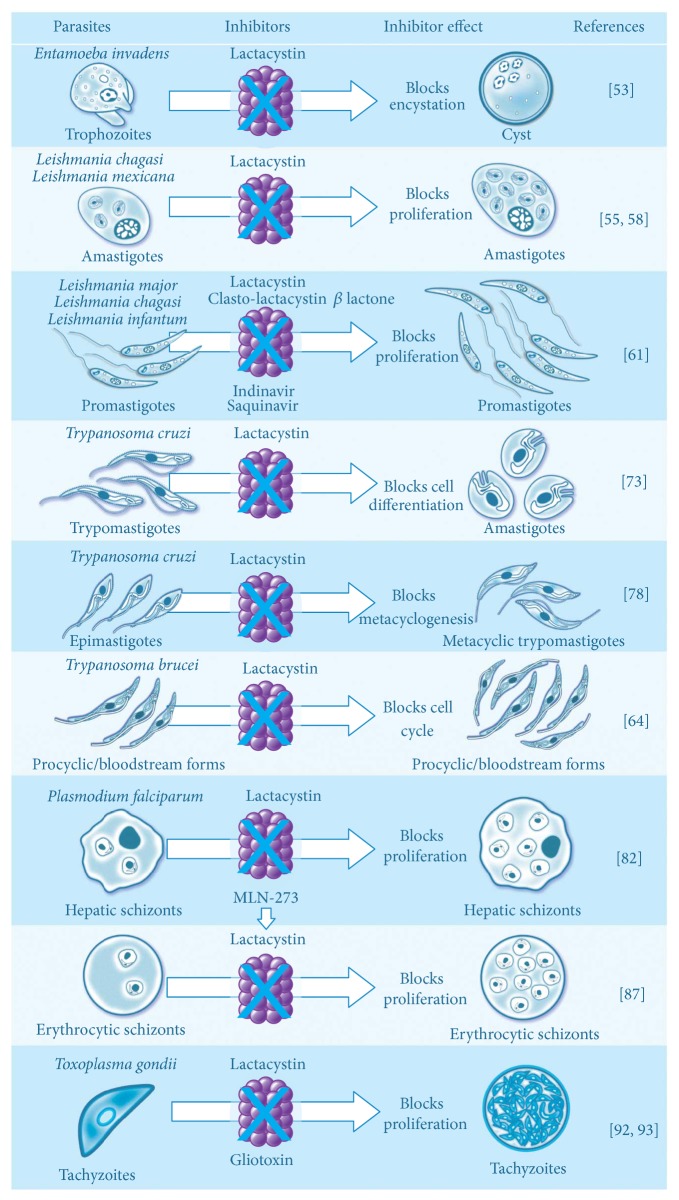
Role of the ubiquitin proteasome system in biology of protozoan parasites and effect of different proteasome inhibitors on proliferation and cell differentiation.

The* Toxoplasma gondii* proteasome has been examined in terms of its intracellular localization and enzymatic activity. Studies of immunofluorescence with antibodies against proteasome have shown that, unlike eukaryotic cells (in which the proteasome is located both in the nucleus and cytosol), in* Toxoplasma,* proteasomes are restricted to the cytosol. Chymotrypsin-like activity was detected, with* Km* values close to those observed in eukaryotic cells [91]. The pretreatment of free tachyzoites with proteasome inhibitors (10 *μ*m lactacystin) or 5 *μ*m gliotoxin [92] did not block the entry of the parasite or the formation of the parasitophorous vacuole but did block intracellular parasite growth and DNA synthesis. However, Shaw et al. [93] showed that lactacystin (2 *μ*m) did not block parasite entry or the establishment of the parasitophorous vacuole but did inhibit parasite growth and daughter-cell budding, as well as DNA synthesis. Pretreatment of host cells with lactacystin did not block parasite entry or development. These results highlight the possible role of* Toxoplasma* proteasome activity in intracellular development and regulation of parasite replication. In the same way, parasite penetration of host cells was not modified by a high gliotoxin concentration (1 *μ*m), but replication was markedly decreased (approximately 50% inhibition by 0.5 *μ*m gliotoxin). Gliotoxin reduced the chymotrypsin-like activity of the* Toxoplasma* proteasome with five times lower potency than in HeLa cells [92]. The major findings about the role of proteasomes in protozoan parasites are summarized in Figure 2.

## 4. Concluding Remarks

Protease activity is essential to many biological systems and processes. In parasites, proteases are essential for host-tissue degradation, immune evasion, and nutrition acquisition [94]. Until less than twenty years ago, the presence and biological role of proteasomes in parasites was not known.

Since the first report of proteasomes in protozoa [63], the first description of its biological function [73], and the first description of the existence of the 26S proteasome in protozoa [74], several reports have confirmed the role of proteasomes in parasite biological processes such as differentiation, the cell cycle, proliferation, and encystation. Proliferation and differentiation are key steps in host colonization. Considering the importance of proteasomes in both processes in many different parasites such as* Trypanosoma*,* Leishmania*,* Toxoplasma,* and* Entamoeba*, parasite proteasomes might serve as virulence factors.

Despite the many parasitic biological phenomena in which the proteasome participates, information relating to how proteasome participates in such phenomena is not known. The majority of the parasite proteins that are degraded by proteasomes have not been identified. Proteasome targets and biological pathways involving proteasomes in key biological process remain to be clarified.

Proteasome inhibitors have been valuable research tools in cellular biology through the elucidation of important biological processes associated with the ubiquitin-proteasome protein-degradation pathway. The ubiquitin-proteasome system is a privileged pharmacological target for drug development due to the tremendous potential for intervention in multiple pathologies including cancer, neurodegenerative diseases, immune diseases, and infections. The pharmacological potential of the UPS was revealed after the unpredicted success of proteasome inhibitors for the treatment of some hematological malignancies.

Moreover, after US Food and Drug Administration approved bortezomib (Velcade) for the treatment of relapsed multiple myeloma, the proteasome has emerged as a new therapeutic target for diverse pathologies. Drug-discovery programs in academia and the pharmaceutical industry have developed a range of low-nanomolar natural and synthetic 20S-proteasome inhibitors and entered them in human clinical trials as significant anticancer and anti-inflammatory leads. The landscape of proteasome inhibitor-based therapeutics is quickly evolving, with promise beyond clinical oncology, and represents an exciting example of translational medicine.

Several pieces of evidence strongly suggest that the ubiquitin-proteasome pathway is also a viable parasitic therapeutic target. Research in recent years has shown that the proteasome is a valid drug target for sleeping sickness [72, 95, 96]. Although the structure of the trypanosome proteasome resembles that of its mammalian counterpart, the enzyme complexes differ from each other with respect to peptidase activity, substrate specificity, and inhibitor sensitivity. In addition, enzymatic analyses have demonstrated that the trypanosome and mammalian proteasomal functions are particularly sensitive to inhibition of the trypsin-like and chymotrypsin-like activities, respectively [97, 98]. Thus, compounds specifically targeting the trypsin-like activity of the trypanosome proteasome may constitute a basis for rational antitrypanosomal drug development. However, the emergence and spread of* P. falciparum* resistance to existing antimalarials necessitates the search for novel drug targets and chemotherapeutic compounds. Inhibition of the proteasome is a promising strategy to develop novel antimalarial drugs.

Diseases caused by parasites affect hundreds of millions of people worldwide, with devastating health and economic effects; however, parasites have been largely neglected in terms of drug development because they affect poor people in poor regions of the world. Most of the drugs currently used to treat these diseases are decades old and have many limitations, including drug resistance. Proteasomes are a key organelle in parasite biology and virulence and appear to be an attractive new chemotherapeutic target.

## Figures and Tables

**Table 1:** Proteasome subunits composition in diferent species of eukaryotic cells.

Protein complex	Subunits	Systematic nomenclature	Miscellaneous nomenclature		
			Human	Yeast	Trypanosomes
		*α*1	Iota	SCL1, YC7	Tb*α*1
		*α*2	C3	PRE8, Y7	Tb*α*2
		*α*3	C9	PRE9, Y13	Tb*α*3
	*α* Type	*α*4	C6	PRE6	Tb*α*4
		*α*5	zeta	PUP2, DOA5	Tc*α*5, TbPSA5
		*α*6	C2	PRE5	Tcpr29*α*6, Tb*α*6
		*α*7	C8	PRE10, YC1	Tb*α*7
	*β* Type	*β*1	Y, delta	PRE3	Tb*β*1
20S		*β*2	Z	PUP1	Tb*β*2
		*β*3	C10	PUP3	Tb*β*3
		*β*4	C7	PRE1	Tb*β*4
		*β*5	X, MB1, epsilon	PRE2, DOA3	Tb*β*5
		*β*6	C5	PRE7	Tb*β*6
		*β*7	N3, beta	PRE4	Tb*β*7
		*β*1i	LMP2, RING12		
		*β*3i	LMP10, MECL1		
		*β*5i	LMP7, RING10		

	ATPase	RPT1	57, MSS1	YTA3, CIM5	TcS7
		RPT2	54, P56	YTA5, mts2	TcS4
		RPT3	56, Tbp7, P48	YTA2	TcS6
		RPT4	S10b, p42	SUG2, CRL3, PLS1	TcS10b
		RPT5	S6′, Tbp1	YTA1	TcYTA-1
		RPT6	58, p45, Trip1	SUG1, CRL3, CIM3/let1	TcS8
		Rpn1	S2, p97	HRD2, NAS1/mts4	TcRpn1, TbRpn1
		Rpn2	S1, p112	SEN3	TbRpn2
		Rpn3	S3, p58	SUN2	TbRpn3
19S		Rpn5	p55	NA55	TbRpn5
		Rpn6	S9, p44.5	NAS4	TbRpn6
		Rpn7	S10a, P44		TbRpn7
	Non ATPase	Rpn8	S12, p40, MOV 34	NAS3	TbRpn8
		Rpn9	S11, p40.5	NAS7, mts1	TbRpn9
		Rpn10	S5a, MBP1	SUN1, MCB1, pus1	TbRpn10
		Rpn11	S13, Poh1	MPR1, pad1, mts5	TbRpn11
		Rpn12	S14, p31	NIN1/MTS3	
		Rpn13	ADRM1	DAQ1	
		Rpn15	DSS1, SHFM1	SEM1	
